# Supplementary figures and images for: Multi-Omics Analysis of Survival-Related Splicing Factors and Identifies CRNKL1 as a Therapeutic Target in Esophageal Cancer
Source: Genes (Basel). 2025 Mar 27;16(4):379. doi: 10.3390/genes16040379 (PMC12027253; doi:10.3390/genes16040379)

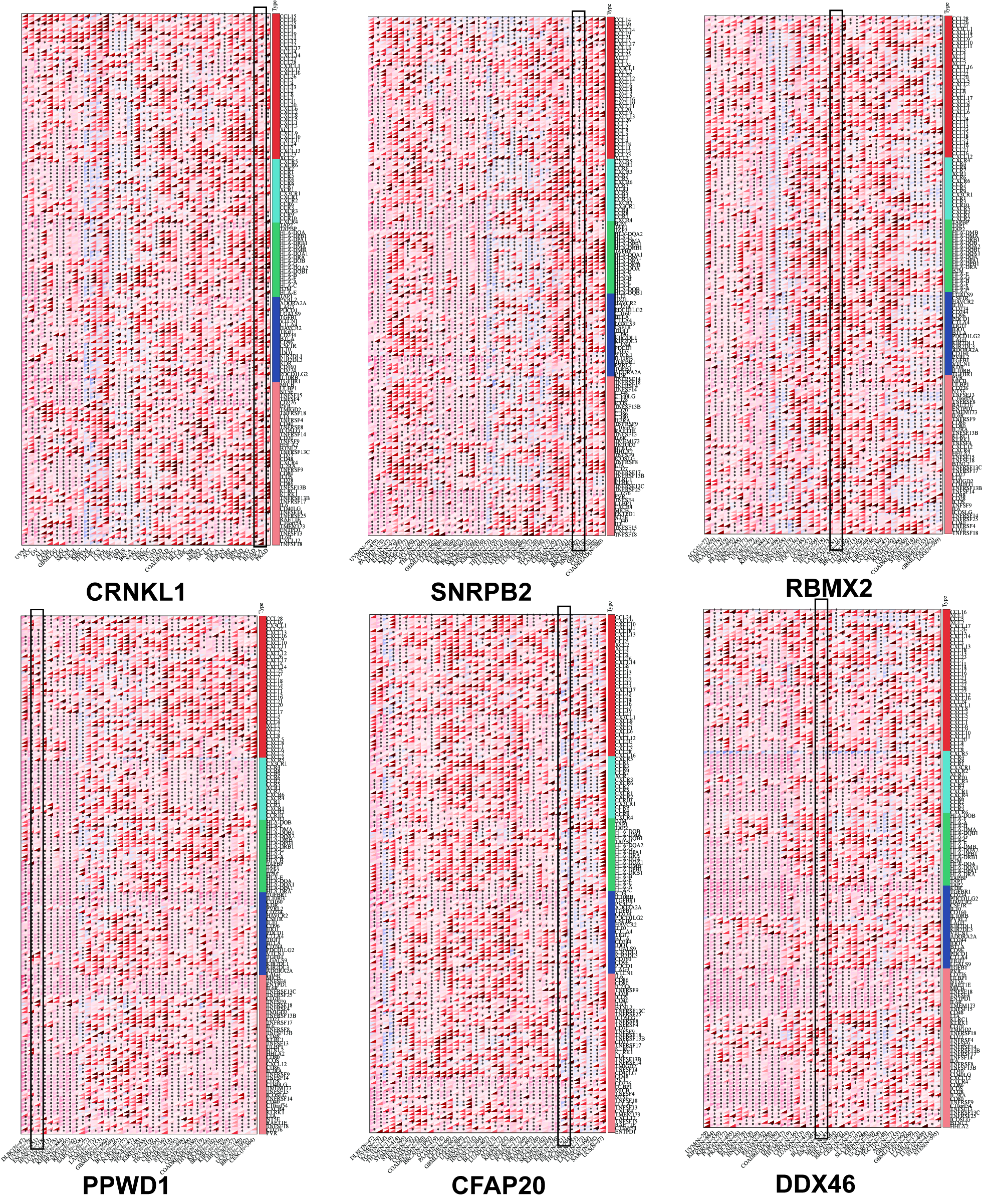

Supplement: Supplementary file 1 [file genes-16-00379-s001.zip › Supplementary Figure S1.tif]

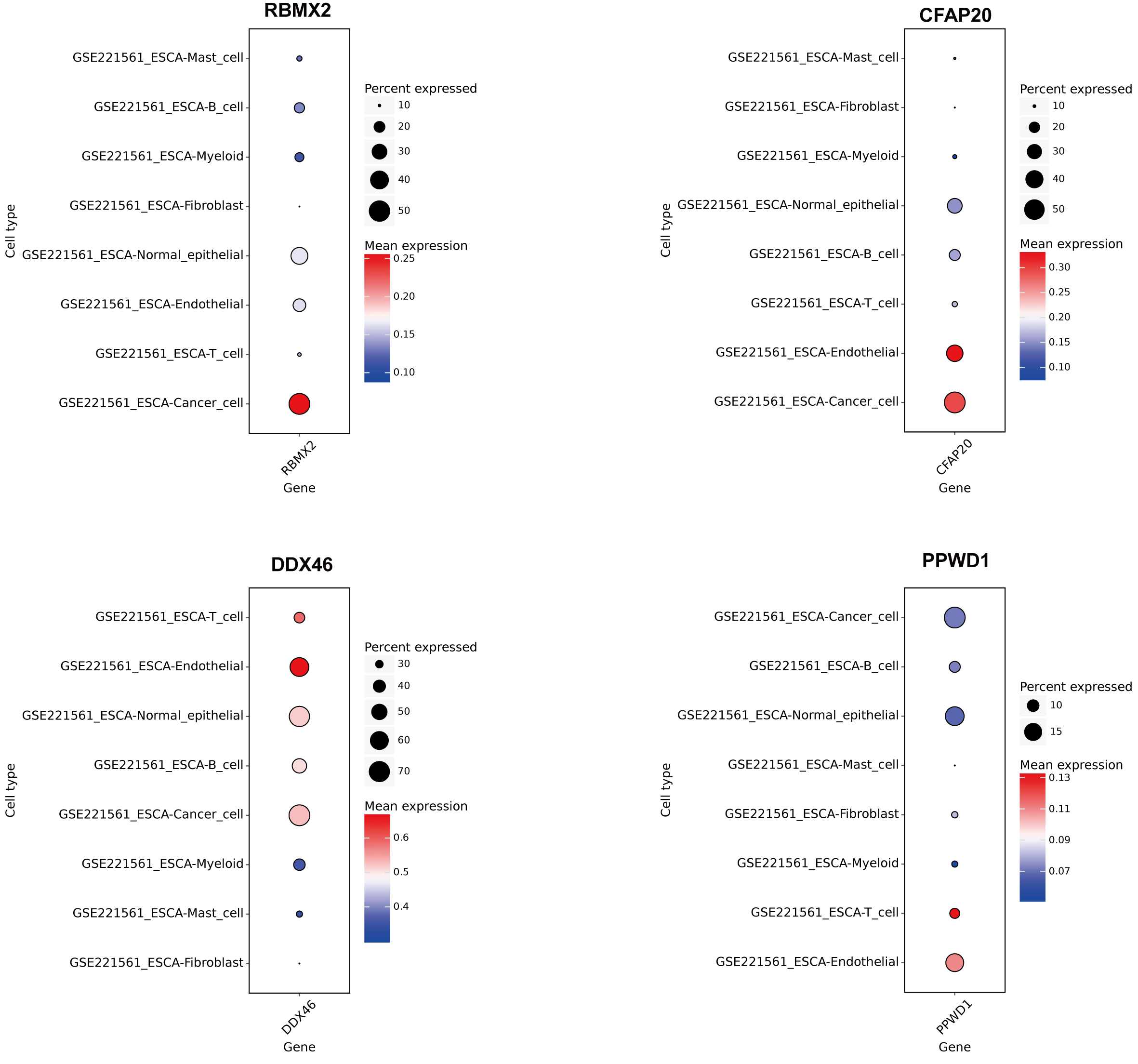

Supplement: Supplementary file 1 [file genes-16-00379-s001.zip › Supplementary Figure S2.tif]

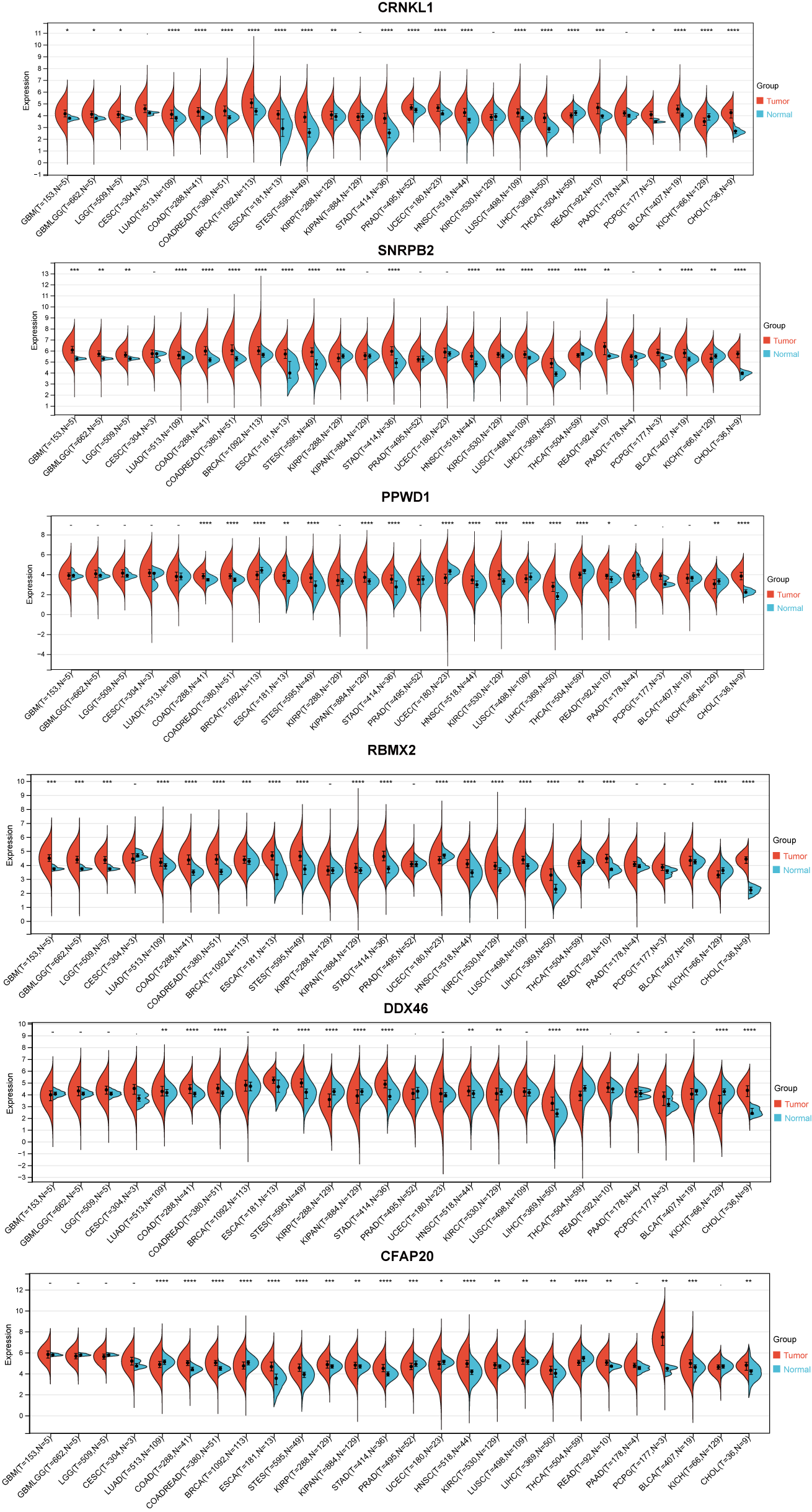

Supplement: Supplementary file 1 [file genes-16-00379-s001.zip › Supplementary Figure S3.tif]

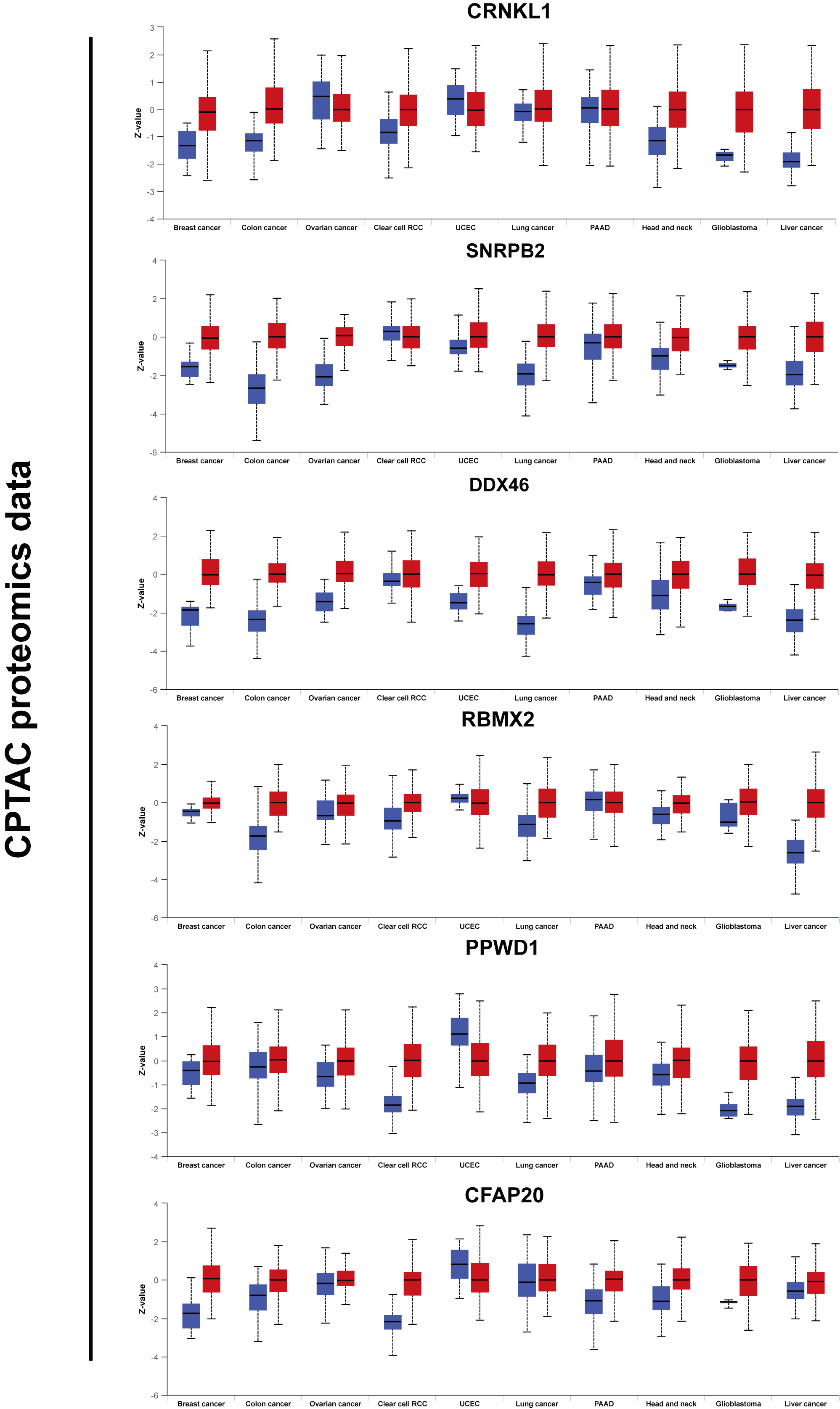

Supplement: Supplementary file 1 [file genes-16-00379-s001.zip › Supplementary Figure S4.tif]

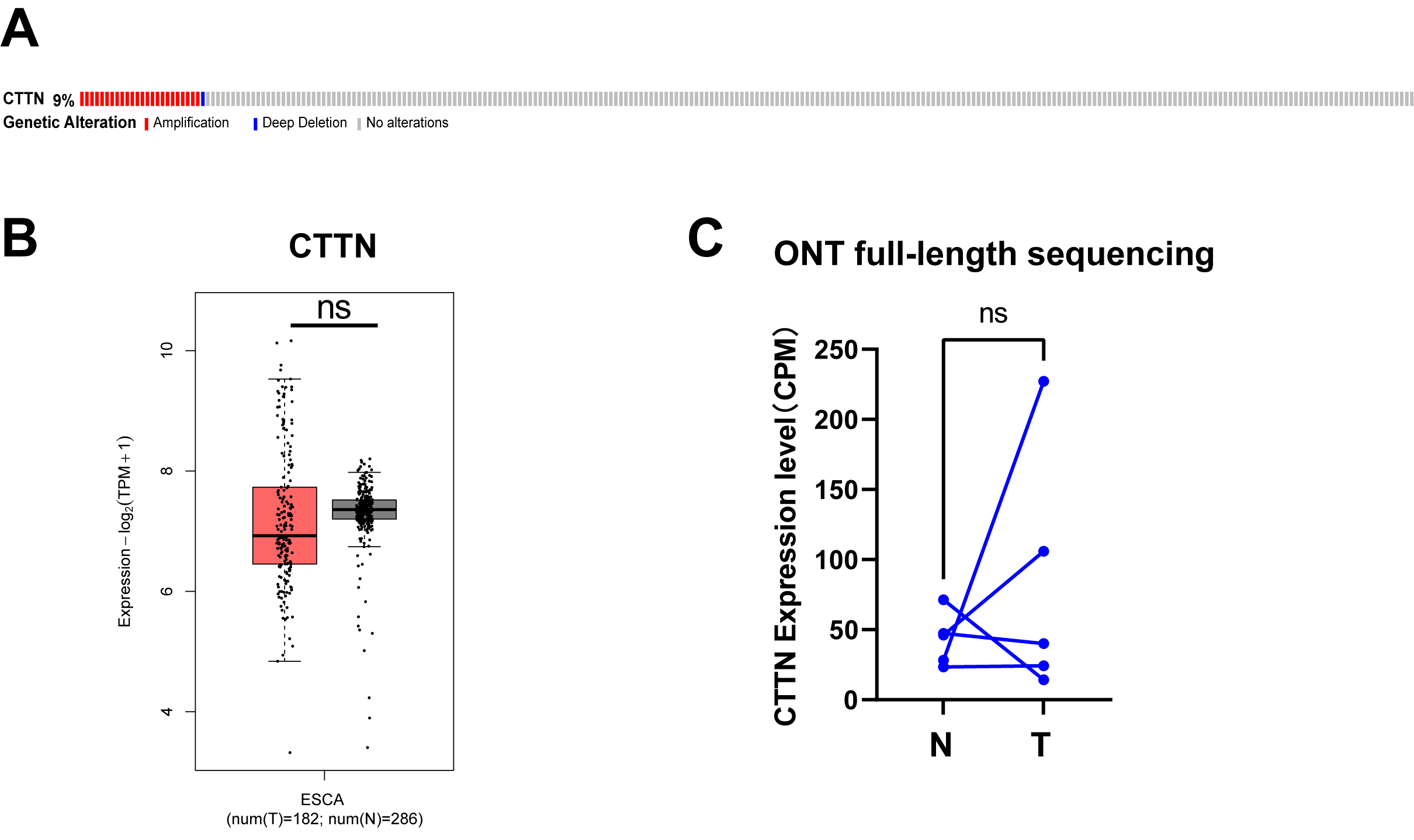

Supplement: Supplementary file 1 [file genes-16-00379-s001.zip › Supplementary Figure S5.tif]
